# Intestinal Behçet's and suspected intestinal Behçet’s disease: a report of four surgical cases

**DOI:** 10.1186/s40792-023-01798-2

**Published:** 2024-01-02

**Authors:** Rika Ono, Tetsuro Tominaga, Takashi Nonaka, Yuma Takamura, Kaido Oishi, Toshio Shiraishi, Shintaro Hashimoto, Keisuke Noda, Terumitsu Sawai, Shinji Okano, Takeshi Nagayasu

**Affiliations:** 1 Department of Surgical Oncology, Nagasaki University Graduate School of Biomedical Science, 1-7-1 Sakamoto, Nagasaki, 852-8501 Japan; 2 Department of Pathology, Nagasaki University Graduate School of Biomedical Science, 1-7-1 Sakamoto, Nagasaki, 852-8501 Japan

**Keywords:** Intestinal Behçet's disease, Surgery, Medication

## Abstract

**Background:**

Intestinal Behçet's disease (BD) is often associated with ulceration that requires surgery, including perforation and abscess formation. However, no consensus has been reached on the optimal extent of resection or treatment strategy. This study reviewed four cases of intestinal or suspected intestinal BD.

**Case presentations:**

In Case 1, a 74-year-old woman diagnosed with BD 2 years earlier was treated with anti-tumor necrosis factor α antibody (Infliximab) and steroids. She had oral and pubic ulcers. After close investigation of abdominal pain, perforation of the gastrointestinal tract was suspected and surgery was performed. Multiple perforating ulcers and abscesses were found in the distal ileum, and the small intestine was resected. Postoperatively, the patient was treated with an increased steroid dose and symptoms have remained stable. Case 2 involved a 69-year-old woman with oral and pubic ulcers, ocular ulcer, and skin lesions. She experienced sudden onset of abdominal pain during treatment for lymphoma. She showed multiple perforating ulcers throughout the ileum and underwent resection of the small intestine and ileostomy. Upper abdominal pain appeared during postoperative treatment for high-output syndrome. The patient underwent omentoplasty after perforation of the upper gastrointestinal tract was diagnosed. Postoperatively, anti-interleukin-1 beta antibodies (canakinumab) was administered to control the disease. Case 3 involved an 81-year-old, previously healthy woman. She presented to her previous physician with complaints of pubic ulcer, hemorrhage and abdominal pain. Colonoscopy showed multiple ulcers throughout the entire colon. Steroid therapy was started, but bleeding proved difficult to control and total proctocolectomy was performed. Histopathology revealed multiple perforating ulcers and BD was diagnosed. Postoperatively, the patient remains under steroid control. Case 4 involved a 43-year-old man with abdominal pain who showed abscess formation in the ileocecal region. After excision of the ileocecal area, multiple ulcers were diagnosed. Two years later, abdominal pain recurred and free air was found in the abdomen on close imaging. Emergency anastomotic resection was performed due to ulceration and perforation of the anastomosis.

**Conclusions:**

Intestinal BD may flare up after surgical treatment and require multiple surgeries. Introducing pharmacotherapy as soon as possible after surgical treatment is important to control the disease.

## Background

Behçet's disease (BD) is a chronic inflammatory immune-mediated disease characterized by recurrent oral and genital ulcers and ocular and dermatological pathologies [1–3]. The prevalence of BD ranges from 0.27 to 5.2 per 100,000 in North America and Scandinavian countries to about 10 per 100,000 in Asia [4]. Patients with BD features who present with ulceration mainly in the ileocecal region and can be differentiated from other etiologies (such as Crohn's disease, intestinal tuberculosis, and drug-induced enterocolitis) are considered to have intestinal BD [5]. The basic treatment for intestinal BD is pharmacotherapy with steroids and anti-tumor necrosis factor α (TNFα) antibodies, but intestinal perforation, bleeding, or stricture often necessitate surgical treatment [6]. Surgery for intestinal BD is associated with a high risk of reoperation (30–44%), but no clear consensus has been reached regarding the appropriate extent of resection or optimal treatment strategy [7–9]. Here, we review four cases of intestinal or suspected intestinal BD at our hospital and discuss the effects of drug therapy and surgery on prognosis.

## Case presentations

Tables 1 and 2 summarize the 6 surgeries conducted for the 4 cases. Patients ranged in age from 43 to 74 years, and 3 patients were female. The underlying disease was myelodysplastic syndrome (MDS) in 1 case, malignant lymphoma in 1 case, and asthma in 1 case, with no underlying pathology in the remaining case. One patient had complete BD with all 4 main signs (oral ulcer, genital ulcer, ocular ulcer, and skin lesion) and 3 cases had incomplete BD. Three of the 6 surgeries were performed for perforation, 2 for abscess formation, and 1 for bleeding. Postoperative complications (high-output syndrome in 1 case; surgical site infection in 1 case) were observed in two patients. The details of each case are described below.

**Table 1:** Clinical characteristics of four patients

Case	Age (years)	Soperaex	Age at onset of BD (years)	Past history	Symptoms of BD						Type of BD
					Oral ulcer	Genital ulcer	Ocular ulcer	Skin lesion	Vascular lesion	Neurologic lesion	
1	74	Female	72	MDS	+	+	−	−	−	−	Suspected
2	69	Female	69	Lymphoma	+	+	+	+	−	−	Complete
3	81	Female	81	Asthma	−	+	−	−	−	−	Suspected
4	43	Male	43	None	+	−	−	−	−	−	Suspected

BD: Bechet’s disease; MDS: myelodysplastic syndrome; Type of BD: according to the 2nd edition of consensus statement for the diagnosis and management of intestinal Bechet’s disease (Ref [5])

**Table 2:** Preoperative characteristics of four patients

Case	Location of disease	Preoperative treatment	Surgical indication	Operation	Postoperative complication	Postoperative treatment	Histopathological findings
1	Ileum	TNFα (Infliximab)/PSL	Perforation	Small bowel resection	None	TNFα (Infliximab)/PSL(dose up)	Multiple ulcers
2–1	Ileum	PSL	Perforation	Small bowel resection, stoma	High-output syndrome	PSL	Multiple ulcers
2–2	Duodenum	None	Perforation	Omentoplasty	None	IL-1β (canakinumab)/PSL	Ulcer
3	Colon	PSL	Bleeding	Proctocolectomy, stoma	Surgical site infection	PSL	Multiple ulcers
4–1	Ileum	None	Abscess	Ileocecal resection	None	PSL	Ulcer
4–2	Anastomotic site	PSL	Perforation, abscess	Resection of anastomotic site, stoma	None	PSL/5-aminosalicylic acid/colchicine	Ulcer

TNFα: anti-TNF alpha antibodies; PSL: prednisolone, IL-1β: anti-interleukin-1 beta antibodies

**Fig. 1:**
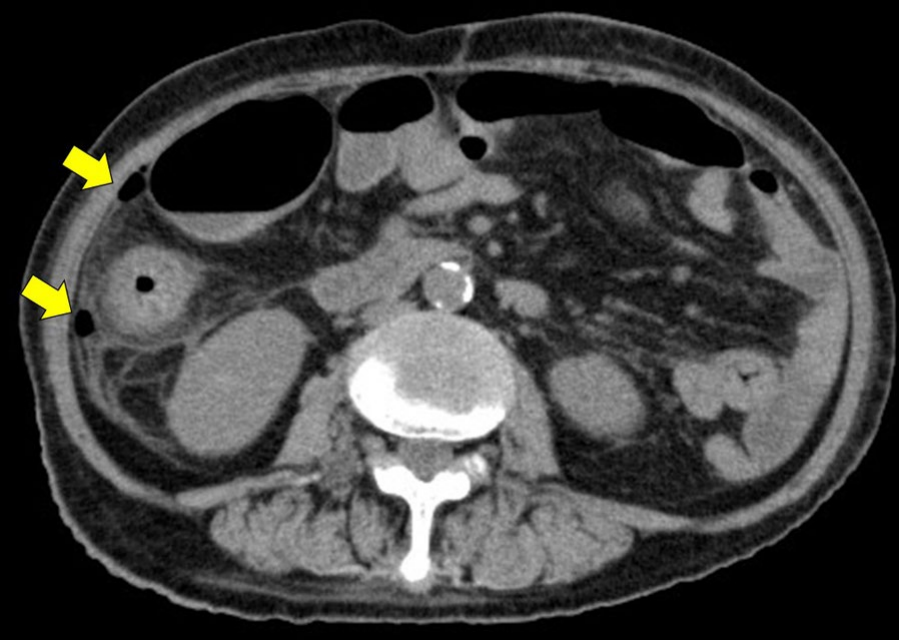
Abdominal CT of Case 1. Wall thickening and free air around the ascending colon are seen. Perforation of the ascending colon was suspected

**Fig. 2:**
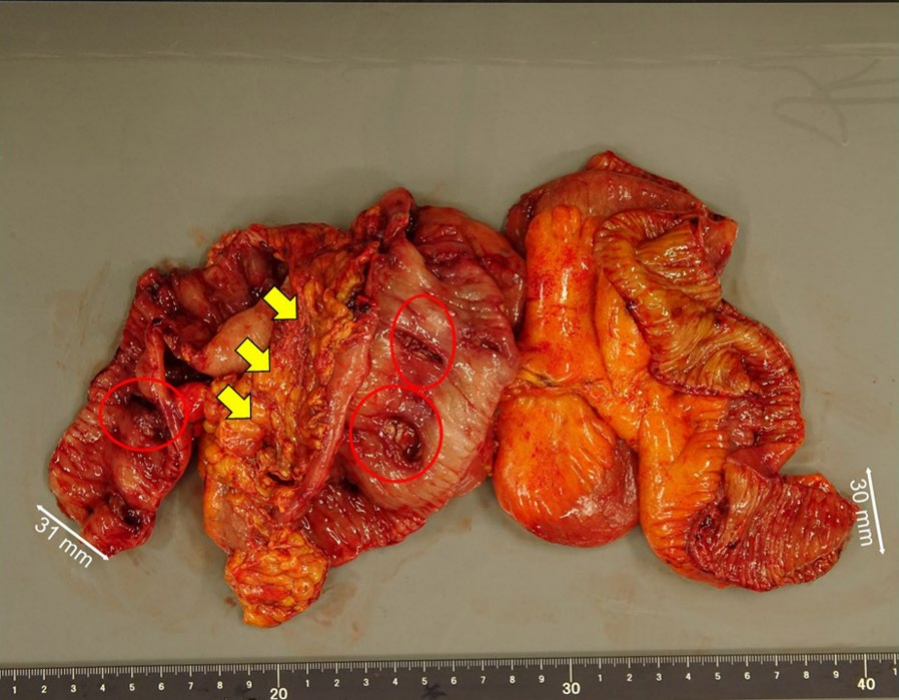
Resected specimen from Case 1. Multiple ulcers (red circle) and abscess (yellow arrow) formation are seen in the ileocecal area

**Fig. 3:**
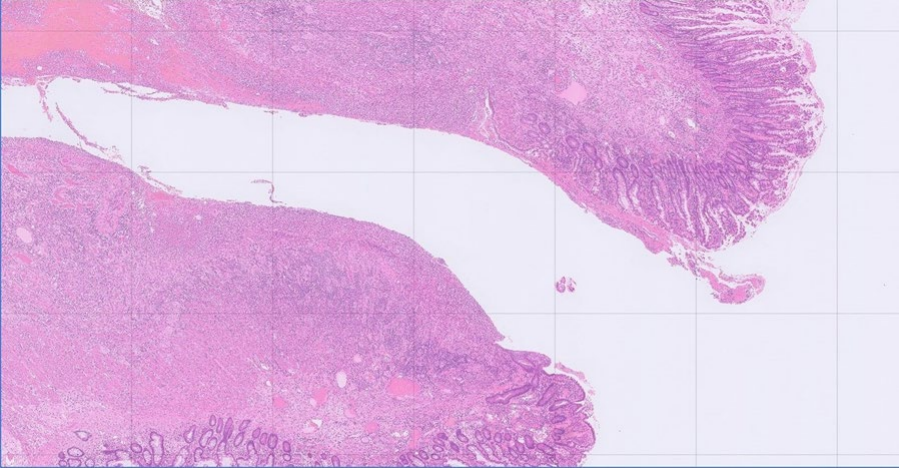
Histological findings for Case 1. The multiple ulcers are consistent with intestinal BD ulceration, as most ulcers reach deep to the subserosal layer

Case 1 involved a 74-year-old woman who had been diagnosed with BD and MDS 2 years earlier and was receiving treatment with anti-TNFα antibody (Infliximab) and steroids. She presented with oral and pubic ulcers. She had experienced sudden abdominal pain and fever around 38 °C, and computed tomography (CT) of the abdomen revealed free air (Fig. 1). Emergency surgery was performed based on a diagnosis of gastrointestinal perforation. Multiple perforating ulcers and abscesses were found in the distal ileum. In addition, partial resection of the small intestine was performed (Fig. 2). The length of bowel resection was 85 cm. Histopathologically, the multiple ulcers were consistent with intestinal BD ulceration, as most ulcers reached deep to the subserosal layer (Fig. 3). The patient continued to receive anti-TNFα antibody (Infliximab) postoperatively and steroid dosage was increased. As of 2 years postoperatively, the intestinal BD remained under control.

Case 2 involved a 69-year-old woman under medical treatment for malignant lymphoma. She had oral, pubic, ocular and skin ulcers. After sudden onset of abdominal pain, CT of the abdomen revealed free air. Emergency surgery was performed under a diagnosis of perforation of the gastrointestinal tract. Multiple perforating ulcers were observed in the ileum, so resection of the small intestine and construction of an ileostomy were performed. The extent of resection was 100 cm considering the perforation site and the condition of the intestinal wall. Finally, the distance to the ileostomy site from the beginning of the jejunum was 120 cm. Histopathological findings showed that there was a nonspecific ulcer seen in Behçet's disease, not malignant lymphoma. After surgery, the patient suffered from high-output syndrome and could not administer the steroid. An upper abdominal pain suddenly appeared 48 days after surgery. Abdominal CT showed free air mainly in the upper abdomen, and the patient underwent reoperation based on the diagnosis of perforation of the upper gastrointestinal tract. Since a perforation was found in the duodenal bulb, omentoplasty was performed. It was determined that the cause was due to intestinal BD, as the perforation was caused by the failure of appropriate drug treatment and BD was uncontrollable, including worsening ocular symptoms and skin ulcers. Thereafter, the patient was treated with steroids and anti-IL-1β antibody (canakinumab). The ileostoma was closed 1.5 years after the initial surgery. As of 2 years postoperatively, the patient has shown no recurrence of intestinal BD.

**Fig. 4:**
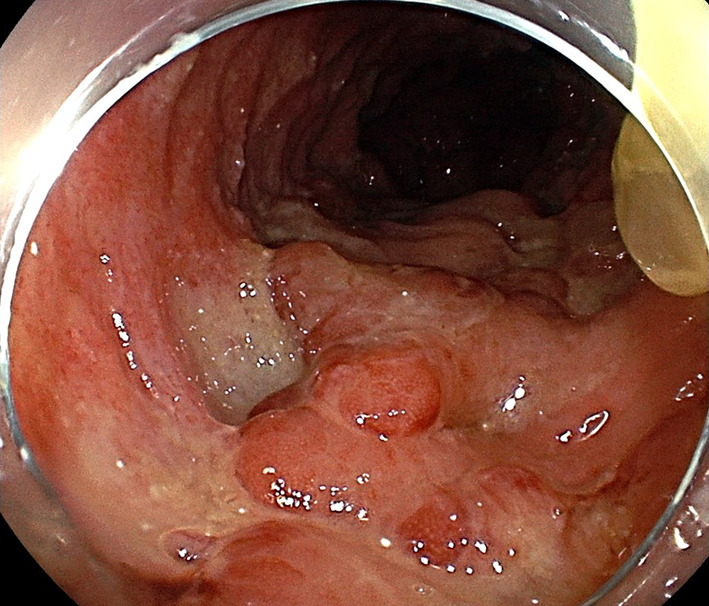
Colonoscopy in Case 3. Colonoscopy reveals multiple ulcers throughout the entire colon

Case 3 was an 81-year-old woman with no previous medical history. Six months earlier, she had developed lower abdominal pain and watery stools, and colonoscopy revealed multiple ulcers throughout the entire colon (Fig. 4). Since she also had a vulvar ulcer, we suspected intestinal BD and started steroids at 30 mg/day, but the hemostasis could not be achieved. Follow-up endoscopy showed that multiple ulcers remained and the patient was judged as difficult to treat medically, so laparoscopic total proctocolectomy plus ileostomy was performed. The length of bowel resection was 130 cm. Histopathology showed multiple perforating ulcers. After surgery, the patient continued to receive steroids and the disease remained well-controlled. As of 4 years postoperatively, no relapses of intestinal BD have been identified.

Case 4 involved a 43-year-old man showing abscess formation in the ileocecal region on CT of the abdomen for close examination of abdominal pain. Emergency ileal resection was performed and pathological examination diagnosed abscess formation due to multiple ulcers. We suspected intestinal BD. Two years later, he revisited our hospital with abdominal pain and underwent anastomotic resection and ileostomy under a diagnosis of retroperitoneal abscess caused by anastomotic ulceration. Thereafter, treatment was initiated with steroids, 5-aminosalicylic acid and colchicine to control the disease. Twenty-six years after disease onset, the ileostomy was closed. No recurrence of intestinal BD has been identified postoperatively.

## Discussion

Intestinal BD is recognized as a special form of BD with ulcerative lesions in the intestinal tract. Although the incidence of gastrointestinal involvement in BD patients varies by region, Japan is reported to show a relatively high incidence of around 50%, and clinicians should be aware of the diagnosis, treatment and follow-up of this pathology [6]. Intestinal BD is said to mainly present with deep, punch-like ulcers with well-defined ulcer margins, showing a predilection (60–75%) for the ileal region [9]. All four cases in the present study also presented with ulcers in the vicinity of the ileocecal area, similar to previous reports.

Pathological features of intestinal BD ulcers include the formation of lymphoid follicles and lymphocytic infiltration around the ulcer, but the periumbilical membrane in the non-ulcer area is reportedly normal [10]. In addition, fissuring from the bottom of the ulcer to the intestinal serosa and weak collagen fiber growth are considered to predispose to perforation [10]. Complications of intestinal BD that require surgery include stenosis, abscess formation, fistula, and intestinal perforation. In previous reports, perforation was the most common complication of intestinal BD (3.3–12.7%) [7]. Of the six surgeries included in this study, four (67%) were perforations of the intestine, likely due to deep ulcers.

When surgery is actually deemed necessary, the optimal extent of bowel resection remains controversial. Chou et al. reported that a greater extent of bowel resection with extended surgery is preferable because of the risk of recurrent ulcers with disease progression [11]. On the other hand, a recent report found no relationship between the length of resection and the recurrence rate, and minimal resection has been recommended [7]. Of the four patients in the present study, those with multiple ulcers underwent minimal resection as necessary. As for the patient who had undergone ileal resection 2 years earlier for ileal abscess, although local resection was performed again due to ulcers causing abscess formation at the anastomotic site.

Some reports have suggested that stoma creation is preferable to temporary anastomosis for patients with intestinal BD because of the high risk of anastomotic leak, perforation, or fistula after surgery due to the general condition of patients and the likelihood of intra-abdominal contamination [12, 13]. However, due to the infrequency of intestinal Behçet's, there is a paucity of literature detailing the causes of the high incidence of anastomotic problems in Behçet's disease. Further case series are needed. In addition, when surgery is required in the chronic stage of intestinal BD, the patient is often treated with steroids, which increases the risk of anastomotic leak, so stoma placement should be considered [12, 13]. Among the six surgeries performed in this study, stoma creation was performed in three cases of steroid therapy and peritonitis. Two cases of them, stoma closure could be achieved at a later date. However, the patient in Case 2 developed high-output syndrome of the ileostomy after the initial surgery and required long-term systemic management. This delayed drug therapy for BD, resulting in aggravation of skin ulcers, ocular ulcers, and gastrointestinal ulcers and BD-related duodenal perforation. The choice of surgical technique thus needs to be considered with care to ensure timely initiation of drug therapy in the early postoperative period.

Intestinal BD is associated with a high reoperation rate of 30–44%, often with the appearance of new lesions at or near the anastomosis [7–9]. Poorly controlled conditions are risk factors [14]. Obtaining control of the disease status of BD early after surgery is important, requiring various drugs [3].

In Case 1, the patient had intestinal BD complicated with MDS. Eighty-seven percent of BD patients with MDS were reported to have trisomy 8, which was associated with poor response to treatment and poor prognosis [15, 16]. Case 1 also had trisomy 8 and developed intestinal BD exacerbation despite ongoing anti-TNFα antibody (Infliximab) and steroid therapy. Adequate drug control was considered important for the refractory patient.

## Conclusions

In this study, we have described four cases of intestinal or suspected intestinal BD that underwent surgical procedures. Appropriate surgical intervention and postoperative pharmacotherapy to control the disease were important in each case.

## Data Availability

The data that support the findings of this study are available from the corresponding author upon reasonable request.

## Abbreviations

BD: Behçet’s disease
TNFα: Tumor necrosis factor-a
MDS: Myelodysplastic syndrome
